# Reconstruction of full-length ureter defects by laparoscopic bladder flap forming

**DOI:** 10.1038/s41598-021-83518-0

**Published:** 2021-02-17

**Authors:** Yuchen Bai, Haibin Wei, Alin Ji, Qi Zhang, Shuai Wang, Yonghan Peng, Xiaofeng Gao, Feng Liu, Dahong Zhang

**Affiliations:** 1 Department of Urology, Zhejiang Provincial People’s Hospital, Hangzhou, 310014 Zhejiang China; 2 Department of Urology, Changhai Hospital, Shanghai, 200433 China

**Keywords:** Urology, Ureter

## Abstract

To evaluate the safety and efficacy of laparoscopic bladder muscle flap reconstruction in the treatment of extensive ureteral avulsion. Patients with full-length (re length > 20 cm) and upper ureteral (avulsion length > 10 cm) defects were eligible. All patients were treated with laparoscopic bladder muscle flap reconstruction. Peri-operative information and post-operative complications were recorded. The kidney function, urinary ultrasound or computed tomography (CT), sun-renal function tests emission computed tomography (ECT) and cystography after operation were recorded. Ten patients were included (7 with full-length and 3 with upper ureteral defects). Median age was 56 years and 70% of them were female. The average operation time and blood loss was 124 min and 92.2 ml. There was no treatment-related adverse effects including urinary leakage, renal colic, fever, etc. The median follow-up was 18.5 months (3–39 months). The surgery did not significantly alter the renal function and separation degree of the renal pelvis during long-term follow-up. Double J stents were removed in nine patients (90%) within six months after operation. Only one case was diagnosed with post-operative anastomotic stricture, and subsequently received laparoscopic ipsilateral nephrectomy one year after the reconstruction operation. All cases had normal voiding and pear-shaped cystography. Laparoscopic bladder flap repair is a safe and effective treatment approach together with several advantages for patients with full-length or upper ureteral avulsion.

## Introduction

With the development and prevalence of ureteroscopic surgery, treatment-related complications have increased substantially in recent years. The incidence of ureteroscopy-related ureteral injury is approximately 3.0–6.7%, depending on the extent, position and time of discovery of the ureteral injuries. The most severe complication is avulsion of the ureter with an incidence of 0.06–0.45%^1–4^.

We used to perform surgical reconstruction for ureteral injuries by using techniques including Boari flap, ileal ureteral substitution, and autologous renal transplantation^5–7^. These methods were effective and successful treatment strategies. The Boari flap is the optimal, but complex, approach for repair of an extensive mid-ureteral injury. The shape of flap is trapezoidal with limited length. This method is primarily suitable for repairing defects in the middle and lower ureter but is not suitable for ureteral defects longer than 15 cm^8^. The open surgery (ileal ureteral substitution and autologous renal transplantation) was relatively complex to conduct with huge trauma. It was difficult to accept and would result in several medical side effects, such as hemorrhage, high risk post-operation infection, longer recover period and so on. To date, there is no ideal strategy to reconstruct the ureter. The main reasons could include the difficulty of open surgery (ileal ureteral substitution and autologous renal transplantation) and the concerns on nephrectomy-related organ loss. The autologous kidney transplantation is a great challenge for those medical centers that do not routinely perform kidney transplantation. Moreover, the replacement of the ureter using intestinal conduit requires removal of the intestinal segment, and this operation bears a relatively high complication rate.

Since 1988, J.P. Blandy et al. reported completed process of Boari flap technique in urological surgery with innovations and continuous controversies. However, considering the limited options for ureteroscopy-related ureteral avulsion and disadvantage of Boari flap, we retrospectively analyzed the clinical data of ten patients treated with laparoscopic bladder muscle flap reconstruction for the treatment of full-length or upper ureteral avulsion. We aimed to investigate the feasibility, efficacy and safety of this surgical procedure. Our results indicated that bladder flap repair under laparoscopy is a safe and effective treatment with several advantages including less trauma, quicker recovery, and fewer complications for patients with full-length or upper ureteral avulsion.

## Materials and methods

### Data collection

We identified patients diagnosed with full-length or upper ureteral avulsion after Iatrogenic ureteral injuries in the Department of Urology, Zhejiang Provincial People's Hospital from November 2014 to September 2019. The major clinicopathological characteristics including age, gender, operation time, blood loss, postoperative complications and time to remove Double J tube were collected. The kidney function, urinary ultrasound or computed tomography (CT), sun-renal function tests emission computed tomography (ECT) and cystography after operation were also recorded.

### Surgical procedure

**Figure 1:**
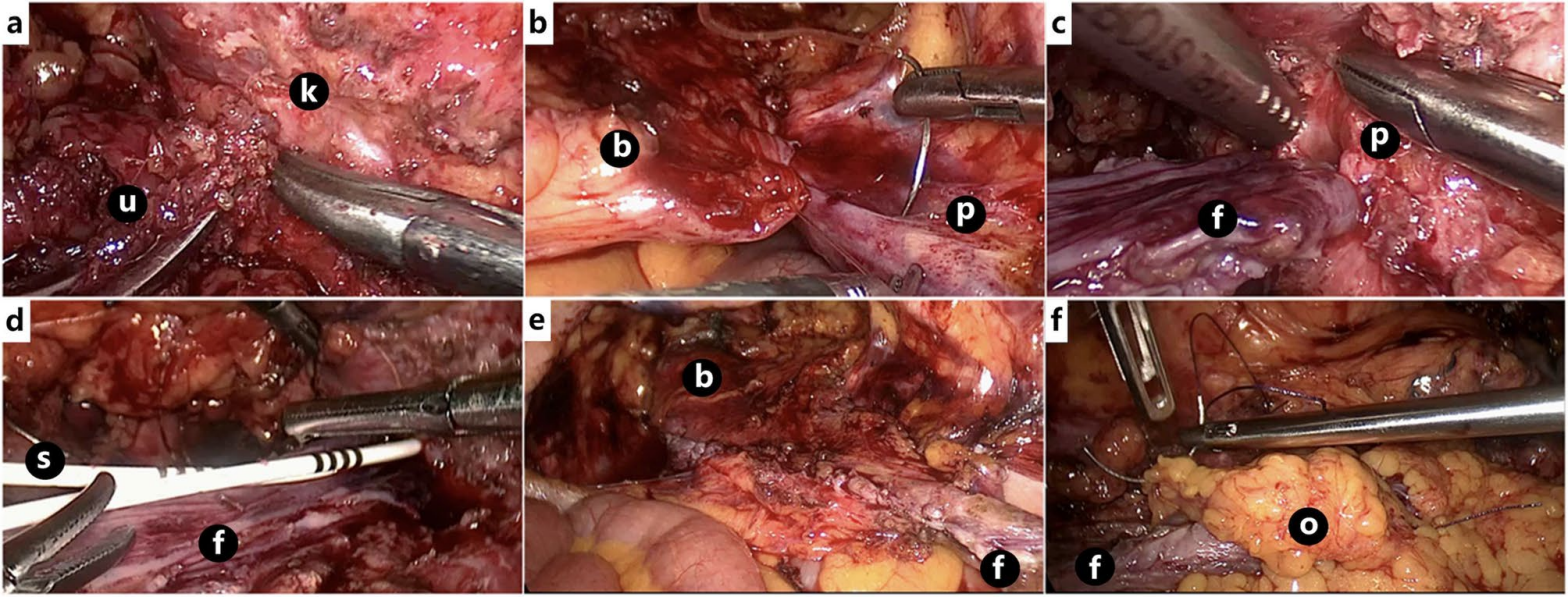
(**a**) separate renal pelvis, upper ureter; (k: kidney; u: ureteral stump), (**b**) psoas major suspension; (b: bladder; p: psoas major), (**c**) inverted bladder flap; (p: pelvis; f: flap), (**d**) double J tube inserted; (s: ureteral stent; f: flap), (**e**) valve tube formation; (b: bladder; f: formation ureter), (**f**) pedicled omentum isolated coverage; (o: great omentum; f: formation ureter).

**Figure 2:**
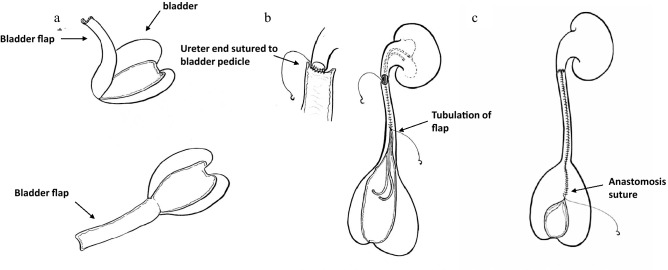
Schematic diagram of the surgery. (**a**) bladder flap taking, (**b**) ureter Reconstruction, (**c**) anastomosis from PUJ to UVJ.

After general anesthesia, lateral decubitus position followed by routine disinfection and draping. First, we opened the anatomical layer, then the affected side of the paracolic sulcus, pushed the affected side of the colon to the interior way in order to expose the surgical field of view at exploration. Second, we isolated renal pelvis and found upper ureter. We separated renal pelvis, upper ureter, and renal inferior pole, and isolated renal pelvis and upper ureter on the dorsal side of the kidney. There was a small amount of hematoma and exudation on the wound surface (Fig. 1a). Third, we cut and overturned the bladder flap, opened the anterior pubic space of the bladder, separated the apex of the bladder and suspended it on the affected side of the psoas major muscle. (Fig. 1b). We measured the distance from the top of the bladder to the renal pelvis. According to this length, we measured the required bladder flap and determined the location of cut, usually from the top to the contralateral ureteral orifice. Electric knife or ultrasonic knife was used to cut bladder flap with a length of 2.0–2.5 cm. Keeping base of it slightly wider when cutting from bladder in order to ensure better blood supply (Fig. 1c). Fourth, valve tube formation: the ureteral stump was sutured to the bladder valve by 4–0 stitches, and two Fr6 double J tubes were inserted (Fig. 1d,e). The remaining bladder flap was sutured into a tubular shape with 4–0 suture then closed the bladder incision with 3–0 suture. At last, pedicle graft of greater omentum: take the pedicled omentum to cover the anastomosis of the upper ureter (Fig. 1f). A drainage tube was placed at each of the pelvic and ureteral anastomosis. The 18F double lumen catheter was indwelled after surgery. Schematic diagram of the surgery was shown in Fig. 2.

The key of our surgical procedure were summaried as follows: 1. Surgical approach and body position: The operation adopts 70–90 degree lateral position of the healthy side and the operation was performed through the abdominal cavity. 2. Search for ureteral stump: it is easy to cause ureteral avulsion due to ureteral mucosal inflammatory edema at the location and increased tissue fragility. 3. Bladder suspension: after the bladder separated, the roof of the bladder was fixed and suspended on the affected side of the psoas muscle to reduce the length of the bladder flap. 4. Selection of bladder muscle flap: the rectangular muscle flap was cut from the bladder wall on the normal side, and the width was about 2.0–2.5 cm. The bottom of the flap is slightly wider in order to maintain rich blood supply. 5. Bladder muscle flap formation: the starting point of the flap and the anastomosis point between the valve and the ureter. In the starting point of the flap, we need to focus on the angle between flap and bladder wall to prevent rotation. We need to pay attention to maintain the enough diameter from suture, because it is easy to result in stenosis and necrosis in this location. Keeping smooth and tension free during the stitching process. For female patient's new ureter, it must pass under the fallopian tube to avoid ureteral compression of the ipsilateral fallopian tube. 6. Protection by pedicled omentum: this is new ureter formed after flap. From anastomosis to flap starting point, blood supply is great enhanced that could reduce the possibility of ischemic necrosis and could improve new ureteral tissue adhesion, rapid absorption of perivascular exudate and establishment of collateral circulation with surrounding tissue.7. Adequate drainage: to avoid stenosis caused by postoperative ureteral stenosis, two DJ tubes were usually inserted for drainage.

### Ethics approval

All procedures performed in studies involving human participants were in accordance with the ethical standards from the Ethics committee of clinical trial institute of Zhejiang provincial people's hospital,and with the 1964 Helsinki Declaration and its later amendments or comparable ethical standards.

### Informed consent

Informed consent was obtained from all individual participants/parents included in the study.

## Results

The demographic and clinical parameters were summarized in Table 1. Among ten eligible patients, 7 of them were females. The median age was 56 years (range 7–72 years). All the operations were successful. The average operation time and hospitalization was 124 min (89–220 min) and 10.5 days (7.0–14.0 days). The average blood loss was 92.2 ml (range 45.0–158.0 ml). No intraoperative blood transfusions were recorded.

**Table 1:** Baseline features of included cases.

Case	Age/gender	Affected side	Avulsion (time)	Avulsion (length)	Nephrostomy (yes/no)	Time of operation (min)	Intraoperative blood loss (ml)	follow-up period (month)	Preoperative affected kidney GFR (ml/min)	Follow-up affected kidney GFR (ml/min)
1	62/male	Right	2 months	15	Yes	117	156	32	27.8	23.6
2	43/male	Right	4 h	21	No	78	45	39	Unidentified	25.6
3	72/female	Right	5 months	18	Yes	130	158	25	21	19.8
4	7/female	Left	2 h	14	No	89	75	37	Unidentified	Unidentified
5	69/female	Right	1 year	20	Yes	145	120	17	27	25
6	68/female	Left	5 h	20	No	220	70	6	Unidentified	Unidentified
7	56/male	Right	1 week	20	No	125	50	4	Unidentified	Unidentified
8	69/female	Left	3 years	20	Yes	120	38	3	38	Unidentified
9	63/female	Right	2 years	21	No	116	80	15	Unidentified	Unidentified
10	49/female	Left	6 months	20	Yes	97	130	7	29	27.6

Six cases with one-stage operation showed no obvious adhesion, which was easy to isolate fistula stump of renal pelvis exit on the dorsal side. One case who has 5 days leakage of urine at post-operation, then we re-operated surgery to remove necrotic ureter and trimmed the renal pelvis due to re-sutured ureter was necrotic. The double J tube was exposed and omentum was unwrapped. Three cases who has obviously adhesive tissue during second-stage operation then ureterostomy was performed. The ureter was difficult to isolate due to sharp isolated ureter renal pelvis and ureter fibrosis, which ureter was useless then separated it from renal pelvis. Two cases with renal fistulation were difficult to separate, hence we isolated it and injected water to distinguish renal pelvis though renal fistulation (one case with ureteral atrophy).

**Figure 3:**
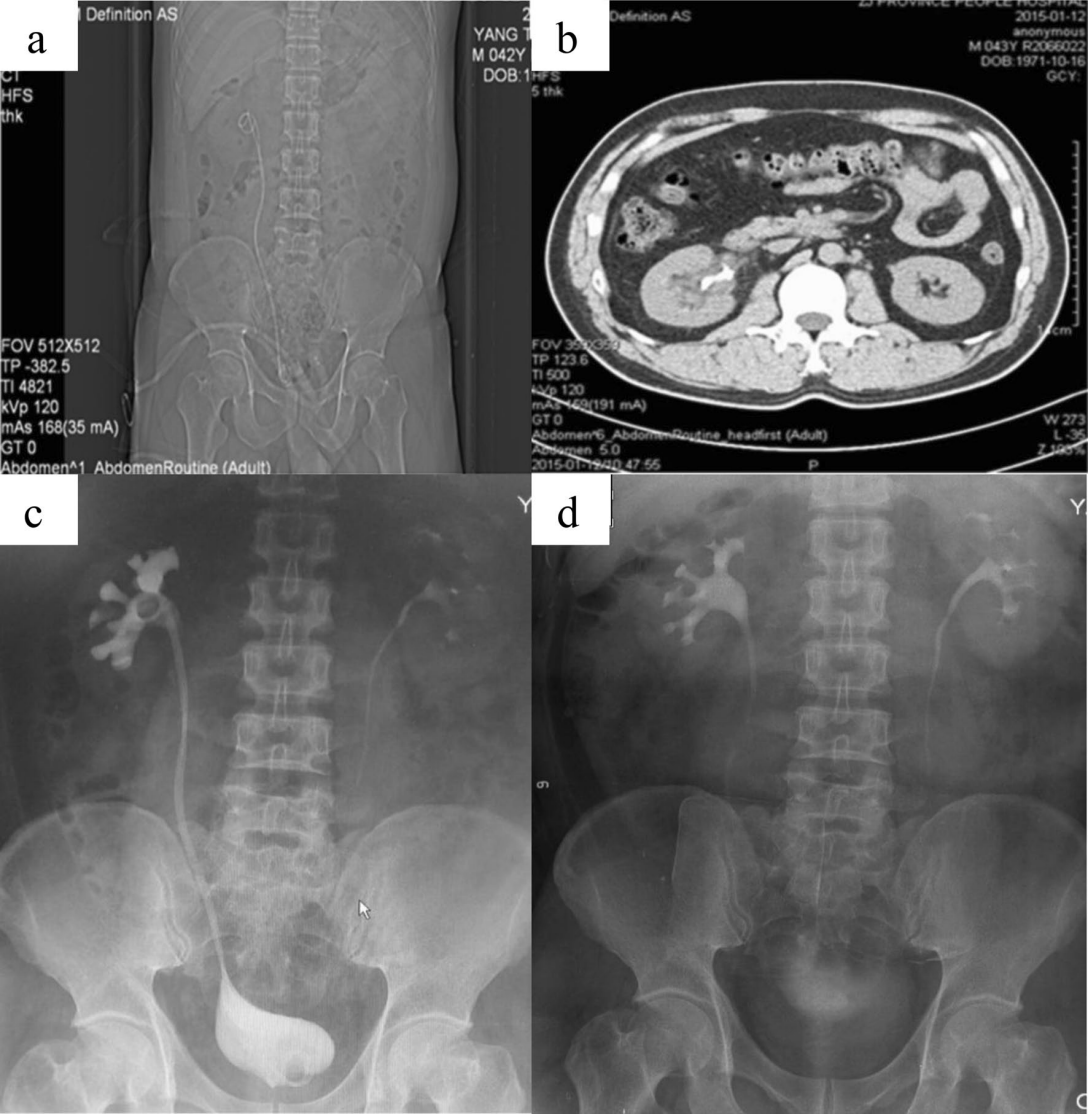
(**a**) patient nr.2 postoperative KUB; (**b**) patient nr.2 postoperative 3 months CT, before double J tube removed; (**c**) patient nr.2 postoperative 3 months IVP with double J tube; (**d**) patient nr.2 postoperative 12 months IVP.

No one had treatment-related complication including leakage of urine, renal colic and high fever during perioperative period. The mean catheter indwelling time was 14 days after surgery. Patient No.2 post-operative KUB, CT and IVP after three and 12 months was shown in Fig. 3. For five patients with avulsion of the upper ureter, their double J tubes were removed three months after operation and two patients with ureter or pyelostomy were replaced with double J tube at three months. The double J tube of one patient was completely removed after six months of operation. Only one case was presented with postoperative back pain and anastomotic stricture. Considering poor residual renal function and frequent requirement of double J tube replacement, laparoscopic ipsilateral nephrectomy was performed after one year of operation. Urinary frequency was not obvious. Cystography showed the pear-shaped bladder. The volume was slightly smaller and the ureteral reflux was not obvious. Postoperative CT showed that renal pelvis was smooth. We did not observe any obviously hydronephrosis and anastomotic stricture in post-operation. During long-term follow-up (range, 3 to 58 months), the patients did not have significant change in renal function and renal pelvis separation.

The regular follow-up examination revealed that three patients with upper-length avulsion and seven patients with full-length avulsion have normal bladder capacity and renal function. Seven of them suffered from full-length ureteral avulsion during ureteroscopic lithotripsy. Six cases received primary emergency operation, including one case with leakage of urine and peritonitis five days after open surgery, and then reperformed with laparoscopic surgery. Two cases were diagnosed with ureteroscopic lithotripsy high avulsion (below the upper ureter), one case with ureteral fistulation for two years and one received post-operative renal fistulation after one year. One case received laparoscopic treatment with left renal cyst decortication and ureterolithotomy due to proximal ureteral calculi three years ago before operation. Ureteroscopy and retroperitoneal laparoscopic ureteral surgery was performed again due to ureteral stricture. Meanwhile, the left ureteral stricture was found during the procedures and end-to-end anastomosis has been done. Nephrostomy was performed because of post-operative leak of urine. The left ureteral orifice was not found during pre-operative cystoscopy. CTU showed left renal pelvic outlet obstruction. Bladder angiography showed the smooth bladder wall and well volume. All patients had normal contralateral renal function and well condition.

## Discussion

Ureteral avulsion, especially the full-length ureteral avulsion, is one of the most devastating but rare complications related to ureteroscopic surgery^9^. Hence, in order to improve the post-operative recover of the iatrogenic ureter ureteral avulsion, we improved the surgical procedure of Boari flap and evaluated the result of laparoscopic bladder muscle flap reconstruction in ten patients with full-length or upper ureteral avulsion after Iatrogenic ureteral injuries.

The common reasons of ureteroscopy-related ureteral avulsion were listed as follows. First, operator would lack of experience of the surgeon, then resulting in improper operative mistake and application of stone baskets or stone pliers. Second, ureteral pathologic conditions included narrow ureteral, inflammatory edema, increased fragility, distorted angulation and so on. Third, condition factors included multiple ESWL, low level spinal anesthesia, inadequate amount of anesthesia, and long operation time, etc.^10,11^. In case of ureteral avulsion, two management principles must be followed: reconstitution of the renal-ureteral-bladder urinary flow pathway and protection of residual renal function. Generally, recommended treatment is autologous kidney transplantation or ileal ureteral anastomosis^12–14^. However, ileal ureteral anastomosis is usually performed by open surgery and requires cutting long segment of small bowel, which would result in huge trauma due to the over complicated surgical procedure (e.g. recovery and reconstruction of the intestine, cutting and anastomosis of the ureteral intestine). We have compared our method with other approached in several publications^15,16^. One previous study ^15^ reported that completely intracorporeal robotic ileal ureteric replacement had longer operative time (328 min), more estimated blood loss (100 ml) and longer length of the ileal substitute (20.4 cm), which was worse results than our method. Moreover, this method required long length of ileal cut which result with huge trauma. The ileal ureteric replacement also required peri-operative gastrointestinal preparation and the patients would have post-operative digestive side-effect.

Previous publications revealed that traditional technique had very high incidence of poor drainage, reflux, azotemia, hyperchloric acidosis, anastomotic stenosis, retro-infection and renal dysfunction^17–20^. Hence, the application of ileal ureteral anastomosis is not desired for this populations. Autologous kidney transplantation was performed under open surgery, the nephrectomy and anastomosis with arteries, veins, and ureters was required. Moreover, some transplanted kidneys have no function due to hydronephrosis, hypertension, renal ischemia–reperfusion injury and so on^12,21,22^(Alizadeh, Valizadeh et al. 2017)]. Therefore, it is very important to develop better surgical methods for the treatment of full-length ureter defects. Recently, bladder flap repair under laparoscope is considered as a novel and useful strategy for ureteral avulsion, especially high or full-length ureteral avulsion injury. Previous boari flap was cut the flap as trapezoidal and over wider bladder flap need to be removed. And boari flap has limited length and is primarily suitable for repairing defects in the middle and lower ureter. It is not suitable for ureteral defects longer than 15 cm. However, our method could overcome these limitations. First, it has minimally invasive effect using laparoscopic surgery. Few trocars decreased abdominal muscle damage as well as better aesthetic dermatology instead of long incisions from open surgery. Hence, acceptability of the operation is very high for both patient and physicians. Second, the tissue comes from the same urinary system. There was no severe treatment-related complications during follow-up period. Moreover, lack of interference with the intestines makes the operation relatively simple. Herein, the operation time is greatly shorter and the trauma is slight. Third, it could protect the kidney function well. If you performed kidney autograft, even if you performed it by greatly kidney function protection way, it will inevitably damage the kidney function. In addition, many patients with ureteral obstruction would have impaired renal function. Finally, it only need 10–12 cm bladder tissue and rest of part was actually completed by translocation of the affected side of the bladder. So the defect segment of the bladder is acceptable and the blood supply of the bladder valve is well preserved. The new ureter contraction and the relative replacement of the anastomosis are well completed. The great omentum wrapping and covering can improve blood supply, reduce leakage of urine and early new ureteral adhesion and fibrosis formation.

There are several disadvantage of our method that should be acknowledged. First, it needs skilled laparoscopic technique team, especially surgeons with adept endoscopic suture techniques. Second, the end of the long segment of the bladder flap is inevitably poorly blood supply. The risk of necrosis and stenosis is relatively high. Third, the cutting of bladder flap is complicated, which could lead to the risk of bladder urination. However, the basic examination of the pre-operative bladder is rarely evaluated especially the health of the bladder wall and the bladder capacity. Hence there is certain degree of blindness for surgeons. Last but not least, the length estimation is challenging, and it requires the normal renal pelvis stump for reconstruction. Thus, it would cause complications including excessive anastomotic tension, anastomosis ischemic and narrow.

At last, we summaries the difference between our method and boari flap. 1. The shape of the flap: Boari's flap is rectangular and our flap is trapezoidal with a broad base. Because the important key point to perform the operation was to maintain the blood supply of the flap, we keep the upper bladder artery on the affected side and the wider base of the flap, which lead to rich blood supply to the distal end and reduce the probability of anastomotic stenosis. 2. The length of the flap: The boari flap was more suitable for middle ureteral avulsion. Our method was better suitable for more distal ureteral avulsion. 3. Difference in surgical methods: In the past, Boari flaps were mostly performed under open surgery. The range of dissociation was relatively large, together with relatively huge damage. The hospitalization and price were also relatively high. Our technology is performed under laparoscopy, which is relatively less traumatic and faster recovery. 4. The safety: We used greater omentum to cover the anastomosis during the operation. The blood supply of the greater omentum could increase the nutrient supply of the anastomosis and reduce the chance of necrosis and stenosis. Furthermore, the anastomosis has been covered by greater omentum. Hence, the anastomotic leakage and urine extravasation seldom happened.

## Conclusion

Bladder flap repair under laparoscopy was a safe and effective treatment with several advantages including less trauma, quicker recovery, and fewer complications for patients with full-length or upper ureteral avulsion. The prospects of this skill is great, but a urologist with rich laparoscopic experience is needed. The long-term efficacy of laparoscopic bladder flap formation and omentum covering still need more clinical data and long-time follow-up. Strictly-designed prospective study with large sample size is also warranted to validate the safety and efficacy of this new surgical approach.
